# A retrospective comparative study comparing laparoscopic and robotic hiatal hernia repair: surgical outcomes, recurrence rate, and postoperative morbidity

**DOI:** 10.1007/s11701-026-03842-3

**Published:** 2026-08-24

**Authors:** Mohammed Humeid, Yusuf Al-Thawadi, Shymaa Salem, Fatima Abdullhafiz, Yaser Janahi, Ahmed Suliman, Ahmed Al Bahrani

**Affiliations:** Hamad Medical Corporation, Doha, Qatar

**Keywords:** Hiatal hernia, Laparoscopic surgery, Robotic surgery, Postoperative morbidity, Hernia recurrence

## Abstract

Hiatal hernia repair is commonly performed using minimally invasive surgical techniques, including laparoscopic and robotic approaches. Although robotic surgery offers enhanced visualization and improved instrument dexterity, controversy remains regarding its superiority over conventional laparoscopy in terms of recurrence, postoperative morbidity, and overall surgical outcomes. This study aimed to compare laparoscopic and robotic hiatal hernia repair regarding surgical outcomes, recurrence rates, and postoperative morbidity. This retrospective comparative study was conducted at Hamad Medical Corporation during the period from January 2019 to September 2025. A total of 126 patients who underwent hiatal hernia repair were included and categorized into laparoscopic and robotic surgery groups according to the operative approach. Data were retrospectively collected from electronic medical records, operative reports, imaging findings, and follow-up documentation. Demographic characteristics, operative details, postoperative complications, recurrence, readmission, reoperation, and mortality outcomes were analyzed. Comparative analysis between both groups was performed using appropriate statistical tests, while multivariable logistic regression analysis was conducted to identify predictors of recurrence and mortality. Statistical significance was considered at *p* < 0.05. A total of 126 patients underwent hiatal hernia repair, including 73 (57.9%) laparoscopic and 51 (40.5%) robotic procedures. The robotic group had a significantly longer mean operative time than the laparoscopic group (4.78 ± 1.25 vs. 3.84 ± 1.36 h, *p* < 0.001), but a significantly shorter mean hospital stay (5.57 ± 1.64 vs. 7.27 ± 3.08 days, *p* < 0.001). Postoperative complications differed significantly between groups, with fewer complications observed in the robotic group (*p* = 0.029). Thirty-day readmission (3.2%) and reoperation (0.8%) were uncommon, with no 30-day postoperative mortality. Overall recurrence occurred in 19.8% of patients and was significantly higher in the laparoscopic group than in the robotic group (27.4% vs. 9.8%, *p* = 0.016). In multivariable analysis, higher ASA score, smoking, and longer operative duration were independently associated with increased recurrence risk, whereas Nissen fundoplication and Phasix mesh reinforcement were associated with lower recurrence odds. Both laparoscopic and robotic hiatal hernia repair demonstrated favorable postoperative outcomes with low rates of major morbidity and mortality. Robotic repair was associated with longer operative duration but showed trends toward lower recurrence and postoperative complication rates. Larger prospective studies are recommended to further evaluate the long-term comparative effectiveness of both surgical approaches.

## Introduction

Even with the benefits offered by laparoscopic surgery, there were a number of technical challenges, including limited range of motion of straight laparoscopic instruments, lack of three-dimensional visualization, surgeon fatigue, and poor camera control during complicated dissection and suturing [1–4]. Robotic assisted surgery was developed to address some of these problems through the use of three-dimensional visualization, tremor filtration, articulation, and dexterity of robotic instruments, and better ergonomics for the surgeon [3–5]. This resulted in increased use of robotic hiatal hernia repair in recent years, especially for complicated and recurrent hiatal hernias.

There have been a number of efforts to compare robotic and laparoscopic hiatal hernia repairs in terms of their perioperative results and postoperative complications. Some studies suggested that robotic surgery produced favorable results, such as reduced length of hospital stay and fewer postoperative complications [1, 5]. Soliman et al. reported less postoperative complications following robotic repair than laparoscopic repair, such as pneumonia, respiratory failure, intensive care unit admissions, urinary tract infection, and surgical site infections [5–7]. In addition, Tolboom et al. noted shorter hospital stay following robot-assisted surgery [1].

On the other hand, other studies have shown concerns about robotic assisted hiatal hernia repairs. Studies by Ward et al. revealed that there were high incidences of complications such as respiratory failure and esophageal perforations among patients who underwent robotic surgery compared to those who had conventional laparoscopy [3]. Issues concerning operative time, recurrence rates, postoperative morbidity, and clinical superiority of robotic over laparoscopic repairs are still being questioned.

Considering the controversial issues surrounding this subject matter and considering the lack of evidence in the literature comparing the two approaches, it is important to conduct a further analysis of the results in regard to surgical outcome, recurrence rate, and postoperative morbidity between laparoscopic and robotic hiatal hernia repair.

## Methods

### Study design

This retrospective comparative study was conducted to evaluate and compare perioperative and postoperative outcomes between patients undergoing laparoscopic versus robotic hiatal hernia repair. The study included consecutive adult patients who underwent elective surgical repair of hiatal hernia at Hamad Medical Corporation between January 2019 and September 2025. Surgical outcomes, postoperative morbidity, and hernia recurrence were assessed and compared between the two surgical approaches.

### Study setting and study period

The study was conducted at Hamad Medical Corporation. Medical records, operative reports, radiological and endoscopic findings, and postoperative follow-up documentation were retrospectively reviewed for patients who underwent hiatal hernia repair between January 2019 and September 2025.

### Study population and patient selection

The study population consisted of adult patients with a confirmed diagnosis of hiatal hernia who underwent elective laparoscopic or robotic hiatal hernia repair during the study period. Patients were categorized according to the surgical approach used, resulting in a laparoscopic repair group and a robotic repair group.

All potentially eligible patients identified from the hospital surgical database during the study period were screened against the predefined eligibility criteria. A total of 126 patients met the eligibility criteria and were included in the final analysis. Consecutive sampling was used, whereby all eligible patients identified during the study period were included.

### Inclusion criteria

Patients were eligible for inclusion if they:

- were aged 18 years or older;
- had a confirmed diagnosis of hiatal hernia;
- underwent elective laparoscopic or robotic hiatal hernia repair;
- had operative and postoperative records sufficient to determine the principal study outcomes; and.
- had at least one documented postoperative follow-up assessment.

### Exclusion criteria

Patients were excluded if they:

- underwent emergency surgery for a strangulated or perforated hiatal hernia;
- underwent open primary repair;
- underwent concurrent major upper gastrointestinal procedures unrelated to hiatal hernia repair;
- had insufficient medical documentation to determine eligibility or the principal outcomes; or.
- had no postoperative assessment after the index operation.

### Sample size and sampling technique

A total of 126 eligible patients were included in the study. A non-probability consecutive sampling approach was used, and all patients meeting the predefined eligibility criteria during the study period were included. As this was a retrospective analysis of all eligible cases available during the predefined study period, no additional sampling was performed.

### Data collection

Data were retrospectively extracted from electronic medical records, operative reports, hospital databases, radiological and endoscopic reports, and follow-up documentation using a structured data collection form developed by the investigators.

Baseline variables included age, sex, body mass index (BMI), smoking status, and relevant comorbidities, including diabetes mellitus, hypertension, and gastroesophageal reflux disease (GERD). Operative variables included operative time, estimated blood loss, type of fundoplication, mesh use, intraoperative complications, and conversion to open surgery.

Postoperative outcomes included length of hospital stay, postoperative pain, pulmonary complications, wound infection, dysphagia, readmission, reoperation, and mortality. Hernia recurrence was determined based on documented postoperative clinical assessment and, when available, radiological or endoscopic evidence. Symptomatic recurrence documented by the treating surgical team was also considered when supported by the medical record.

### Follow-up and assessment of recurrence

Postoperative follow-up was based on the clinical visits and investigations documented in the medical records. Follow-up duration was calculated from the date of the index operation to the date of the last documented postoperative assessment or the date of documented recurrence, where applicable. The duration of follow-up was summarized for the overall cohort and compared between the laparoscopic and robotic groups.

Recurrence was assessed during postoperative follow-up using clinical evaluation, imaging, endoscopy, or documented symptomatic recurrence. Patients without documented recurrence at their last available follow-up were considered recurrence-free up to that documented follow-up date. Patients with no postoperative follow-up documentation were excluded according to the predefined eligibility criteria.

### Missing data

The database was reviewed for completeness and internal consistency before statistical analysis. Variables with missing observations were identified and the number of available observations was reported where applicable. No assumptions were made regarding unrecorded clinical outcomes. For analyses involving variables with missing values, only patients with available data for the respective variable were included. The denominators for categorical and continuous variables were therefore based on the number of available observations for each analysis.

### Outcome measures

The primary outcomes were:

- postoperative hernia recurrence; and.
- postoperative morbidity.

Secondary outcomes included:

- operative time;
- estimated blood loss;
- length of hospital stay;
- conversion to open surgery;
- readmission;
- reoperation; and.
- postoperative mortality.

Postoperative morbidity was defined as any documented postoperative complication occurring after the index operation during the predefined postoperative assessment period and included the complications recorded in the study database, such as pulmonary complications, wound infection, dysphagia, and other clinically documented adverse events.

### Operational definitions

Hernia Recurrence: Hernia recurrence was defined as the reappearance of a hiatal hernia or intrathoracic migration of the stomach or gastroesophageal junction following the index repair, as documented by postoperative imaging, upper gastrointestinal contrast study, computed tomography, upper gastrointestinal endoscopy, or other objective diagnostic investigation. Recurrence documented by the treating surgeon based on clinical assessment and supported by the medical record was also recorded. The date of recurrence was defined as the date on which recurrence was first objectively documented or clinically diagnosed.

Surveillance Strategy for Recurrence: Postoperative surveillance was based on routine clinical follow-up documented in the medical records. Patients were assessed for symptoms suggestive of recurrence, including recurrent reflux, dysphagia, chest or epigastric discomfort, regurgitation, or other recurrent upper gastrointestinal symptoms. Diagnostic investigations, including upper gastrointestinal contrast studies, computed tomography, and/or upper gastrointestinal endoscopy, were performed when clinically indicated or when recurrence was suspected. Recurrence was recorded when supported by objective imaging/endoscopic evidence or by a documented clinical diagnosis by the treating surgical team. Patients without documented recurrence were considered recurrence-free up to their last available postoperative assessment.

Duration of Follow-up: Follow-up duration was calculated from the date of the index hiatal hernia repair to the date of the last documented postoperative clinical assessment, the date of documented recurrence, or the date of death, whichever occurred first. Follow-up duration was summarized separately for the laparoscopic and robotic groups using the mean ± standard deviation or median and interquartile range, as appropriate. Patients with no documented recurrence were considered recurrence-free for the duration of their available follow-up.

Readmission: Readmission was defined as an unplanned hospital admission occurring after discharge from the index hospitalization and within the predefined postoperative follow-up period, when the admission was related to the index hiatal hernia repair or a postoperative complication. Admissions unrelated to the index surgical procedure were not considered surgical readmissions.

Postoperative Mortality: Postoperative mortality was defined as death occurring after the index hiatal hernia repair and before completion of the postoperative follow-up period, when documented in the medical record. The date and documented cause of death were recorded when available. Mortality was considered a postoperative outcome regardless of whether death occurred during the index hospitalization or following discharge, provided that it was documented within the predefined postoperative follow-up period.

### Statistical analysis

Statistical analyses were performed using the Statistical Package for the Social Sciences (SPSS), version 23 (IBM Corp., Armonk, NY, USA). Continuous variables were assessed for distribution and are presented as mean ± standard deviation (SD) for approximately normally distributed data or median with interquartile range (IQR) for non-normally distributed data. Categorical variables are presented as frequencies and percentages.

Baseline characteristics and perioperative outcomes were compared between the laparoscopic and robotic groups. The independent-samples t test was used for normally distributed continuous variables, whereas the Mann–Whitney U test was used for non-normally distributed continuous variables. Categorical variables were compared using the Pearson chi-square test or Fisher’s exact test when expected cell counts were small.

Univariable logistic regression analyses were performed to assess the association between potential explanatory variables and the primary binary outcomes of postoperative recurrence and postoperative morbidity. Variables considered clinically relevant and/or demonstrating an association with the outcome in univariable analysis were considered for inclusion in multivariable logistic regression models. The final models were constructed while considering the number of outcome events to reduce the risk of model overfitting. Adjusted odds ratios (ORs) with 95% confidence intervals (CIs) were reported.

The surgical approach (laparoscopic versus robotic) was included as the principal exposure variable in the multivariable analyses, with adjustment for relevant baseline and clinical covariates where appropriate. Multivariable analyses were interpreted as estimates of association rather than causal effects, given the retrospective observational design of the study. A two-sided p value < 0.05 was considered statistically significant.

### Ethical considerations

Ethical approval for the study was obtained from the Institutional Review Board of the Medical Research Center Team, Hamad Medical Corporation, under approval number MRC-01-25-1084. Patient confidentiality and privacy were maintained throughout the study. Data were anonymized before analysis and used solely for research purposes. Given the retrospective nature of the study, the requirement for individual informed consent was waived by the ethics committee.

## Results

### Baseline characteristics of the study population

A total of 126 patients who underwent hiatal hernia repair were included in this retrospective comparative study. The mean age of the study population was 49.4 ± 15.33 years, with a median age of 48 years. Most patients were middle-aged adults, with the largest proportions clustered between 30 and 60 years of age. Regarding sex distribution, males represented 50.4% (*n* = 63) of the cohort, while females accounted for 49.6% (*n* = 62). One patient had missing gender documentation.

The mean body mass index (BMI) was 30.82 ± 6.17 kg/m², indicating that the majority of patients were overweight or obese. Smoking history revealed that 28% (*n* = 35) were active smokers.

Comorbid diseases were highly prevalent among the included patients. Although 37.3% (*n* = 47) had no documented comorbidities, the remaining patients demonstrated a broad spectrum of chronic medical conditions. Hypertension, diabetes mellitus, hyperlipidemia, asthma, hypothyroidism, obstructive sleep apnea, chronic kidney disease, and coronary artery disease were among the most frequently encountered comorbid illnesses. Combined comorbidity profiles were also common, particularly hypertension with diabetes mellitus (Table 1).

**Table 1:** Demographic and Clinical Characteristics of the Study Population (*N* = 126)

Variable		Surgical Approach		*P* value
		Laparoscopic	Robotic	
Age, mean ± SD (years)		48.09 ± 16.61	50.43 ± 14.84	0.420
BMI, mean ± SD (kg/m²)		30.38 ± 6.46	30.85 ± 7.17	0.703
Gender	Male	36	27	0.386
	Female	38	24	
Smoking	Yes	22	13	0.378
	No	52	38	
Presence of comorbidity		46	33	0.423

### Presenting symptoms and preoperative findings

The majority of patients presented with symptomatic gastroesophageal reflux disease (GERD)-related manifestations. Reflux/GERD, dysphagia, regurgitation, epigastric pain, cough, vomiting, aspiration symptoms, and iron-deficiency anemia were among the most common presenting complaints. Complex symptom combinations were frequently reported, reflecting the multifactorial clinical presentation of hiatal hernia disease.

The duration of symptoms varied substantially among patients. The most frequently reported symptom duration was 2 years (11.1%), followed by 1 year (8.7%), 3 years (7.9%), and 5 years (7.1%). A small subset of patients presented acutely with symptoms developing within days or weeks.

Cameron ulcers were identified in 3.2% (*n* = 4) of cases, whereas Barrett’s esophagus was documented in 3.2% (*n* = 4). Among patients with Barrett’s esophagus, the recorded Barrett’s segment lengths ranged from 3 to 6 cm.

The mean axial hernia length was 3.29 ± 2.58 cm. Type I sliding hiatal hernia represented the most common anatomical subtype, accounting for 35.7% (*n* = 45) of patients, followed by Type III mixed hernias (19.8%, *n* = 25), Type II paraesophageal hernias (9.5%, *n* = 12), and Type IV gastric incarceration hernias (6.3%, *n* = 8) (Table 2).

Los Angeles esophagitis grading demonstrated that 35.7% of patients had normal findings, whereas Grade B esophagitis was the most common abnormal grade (17.5%). Preoperative manometry was normal in 31.0% of patients, while ineffective esophageal motility was reported in 11.9%.

**Table 2:** Preoperative Clinical and Endoscopic Findings

Variable		Surgical Approach		*P* value
		Laparoscopic	Robotic	
Cameron Ulcer		4	0	0.234
Esophagitis Grade	Normal	50	25	0.113
	A	8	9	
	B	13	9	
	C	2	6	
	D	1	2	
Barrett Esophagus		0	4	0.046
Hernia Type	Sliding	24	21	0.019
	Para-esophageal	10	2	
	Mixed	12	13	
	Gastric incarceration	8	0	
Preoperative Manometry	Normal	18	21	0.767
	IEM	7	8	
	Outflow obstruction	1	1	
	Achalasia/Others	1	0	

### Operative characteristics

Most operations were performed electively (84.9%, *n* = 107), while 10.3% (*n* = 13) were semi-elective and 4.0% (*n* = 5) were emergency procedures.

The laparoscopic approach represented the predominant operative technique and was utilized in 57.9% (*n* = 73) of patients, whereas robotic surgery was performed in 40.5% (*n* = 51). One laparoscopic procedure required conversion to open surgery.

Nissen fundoplication was the most frequently performed anti-reflux procedure (37.3%), followed by Dor fundoplication (31.7%) and Toupet fundoplication (19.0%). No fundoplication was performed in 8.7% of patients.

Regarding crural closure materials, barbed non-absorbable V-Loc sutures were the most commonly used (54.8%), followed by braided polyester Ethibond sutures (24.6%). Mesh reinforcement was not utilized in 24.6% of cases, whereas Phasix ST mesh represented the most frequently used mesh material (25.4%). Tissue glue was used in 29.4% of procedures (Table 3).

The mean operative duration was 4.23 ± 1.39 h, ranging from 1 to 8.5 h. Most procedures lasted between 3 and 5 h.

**Table 3:** Operative Characteristics

Variable		Surgical Approach		*P* value
		Laparoscopic	Robotic	
Elective surgery	Elective	58	49	0.019
	Semi-elective	12	1	
	Emergency	4	1	
ASA score	I	4	6	0.355
	II	45	32	
	III	23	13	
	VI	2	0	
Fundoplication Type	Nissen	18	29	< 0.001
	Toupet	10	14	
	Dor	34	6	
Crural suture	Polyester	34	15	0.005
	Tricron/monofilament	7	0	
	V-Loc	33	36	
Mesh Material	None	22	9	< 0.001
	Phasix ST	16	16	
	Gore BIO-A	4	0	
	Ovitex	5	18	
	ePTFE Patches	13	0	
	Permacol	4	1	
	Others	10	7	
Tissue Glue		14	23	0.002
Postoperative Complications	No complications	41	39	0.029
	Grade I	13	9	
	Grade II	9	0	
	Grade IIIa	7	1	
	Grade IIIb	2	0	
	Grade Via	2	2	
Pleural Breach		15	6	0.157

### Postoperative outcomes and morbidity

The mean postoperative hospitalization duration was 6.57 ± 2.72 days, with hospital stays ranging from 3 to 20 days. ICU admission was uncommon, with most patients (88.0%) not requiring intensive care support.

Pleural breach represented one of the most frequently encountered intraoperative complications and occurred in 16.7% (*n* = 21) of cases. Postoperative complications according to Clavien–Dindo classification demonstrated that 63.5% of patients experienced no postoperative complications. Grade I complications occurred in 17.5% of patients, while Grade II complications were documented in 7.1%. More severe complications requiring intervention or ICU admission were less common.

Complication timing analysis demonstrated that most adverse events occurred during the intraoperative or immediate postoperative period. Pleural injuries, pneumothorax, dysphagia, surgical emphysema, respiratory complications, wound infections, urinary retention, and gastrointestinal symptoms were among the documented postoperative complications (Table 4).

During follow-up, dysphagia was the most frequently reported persistent symptom, followed by pain, reflux symptoms, diarrhea, bloating, nausea, and vomiting.

**Table 4:** Postoperative Outcomes and Complications

Variable		Surgical Approach		*P* value
		Laparoscopic	Robotic	
Fundoplication Type	Nissen	18	29	< 0.001
	Toupet	10	14	
	Dor	34	6	
Postoperative Complications	No complications	41	39	0.029
	Grade I	13	9	
	Grade II	9	0	
	Grade IIIa	7	1	
	Grade IIIb	2	0	
	Grade Via	2	2	
Pleural Breach		15	6	0.157
Need for ICU admission		13	3	0.049
Mean procedure duration (hours)		3.84 ± 1.36	4.78 ± 1.25	< 0.001
Mean hospitalization duration (days)		7.27 ± 3.08	5.57 ± 1.64	< 0.001

### Readmission, reoperation, and recurrence

Thirty-day readmission was uncommon and occurred in only 3.2% (*n* = 4) of patients. Reasons for readmission included postoperative vomiting, dehydration, constipation, abdominal pain, respiratory complications, and pneumonia.

Only one patient (0.8%) required reoperation within 30 days postoperatively.

Hiatal hernia recurrence was identified in 19.8% (*n* = 25) of patients during follow-up. Most recurrences occurred within the first postoperative year, accounting for 76.0% of all recurrence cases. Smaller proportions recurred after 2, 3, or 5 years (Table 5).

No 30-day postoperative mortality was observed in the study cohort.

**Table 5:** Readmission, Reoperation, and Recurrence Outcomes

Variable	Surgical Approach		*P* value
	Laparoscopic	Robotic	
30-day readmission	1	3	0.181
30-day reoperation	0	1	0.405
Recurrence	20	5	0.016
Mortality	0	0	-

### Comparative analysis between laparoscopic and robotic repair

The multivariable logistic regression analysis identified several independent predictors associated with postoperative hiatal hernia recurrence (Table 6). Patients with higher ASA scores (III–IV) demonstrated significantly increased odds of recurrence compared with those with lower perioperative risk profiles (OR = 11.6, *p* = 0.003). Emergency surgical intervention was also strongly associated with recurrence risk (OR = 7.54, *p* = 0.022), indicating the negative impact of urgent presentation and limited preoperative optimization. Severe postoperative complications (Grade III–IV) and intraoperative pleural breach were additional major predictors, increasing recurrence risk by more than sixfold and fivefold, respectively. Smoking independently increased recurrence risk approximately 2.4 times, further emphasizing the role of patient-related risk factors in long-term surgical durability.

Regarding operative techniques, Nissen fundoplication demonstrated a significant protective effect against recurrence compared with no fundoplication (OR = 0.33, *p* = 0.035), whereas Dor and Toupet fundoplication did not achieve statistical significance after adjustment. V-Loc suture use was associated with significantly lower recurrence risk (OR = 0.39, *p* = 0.039), while Ethibond suture use showed no independent effect. Among reinforcement materials, Phasix mesh significantly reduced recurrence risk compared with no mesh use (OR = 0.36, *p* = 0.043), whereas Ovitex mesh did not demonstrate statistical significance. Tissue glue use also showed a protective association against recurrence (OR = 0.44, *p* = 0.049). Additionally, prolonged operative duration was independently associated with higher recurrence risk, suggesting that technically complex procedures may contribute to long-term failure.

**Table 6:** Multivariable Logistic Regression Analysis of Predictors of Postoperative Recurrence

Predictor	B	SE	Wald	*p*-value	OR (Exp(B))	95% CI
ASA Score (III–IV vs. I–II)	2.45	0.81	9.12	0.003	11.60	2.4–55.1
Smoking (Yes vs. No)	0.89	0.44	4.10	0.043	2.43	1.02–5.78
Nissen Fundoplication vs. No Fundoplication	-1.12	0.53	4.47	0.035	0.33	0.12–0.91
Dor Fundoplication vs. No Fundoplication	-0.76	0.49	2.41	0.121	0.47	0.18–1.25
Toupet Fundoplication vs. No Fundoplication	-0.58	0.52	1.24	0.265	0.56	0.20–1.59
Phasix Mesh vs. No Mesh	-1.03	0.51	4.08	0.043	0.36	0.13–0.97
Ovitex Mesh vs. No Mesh	-0.68	0.54	1.59	0.207	0.51	0.17–1.54
Tissue Glue Use (Yes vs. No)	-0.81	0.43	3.55	0.049	0.44	0.19–0.99
Procedure Duration (hours)	0.41	0.18	5.19	0.023	1.51	1.06–2.16

The multivariable logistic regression model (Table 7) evaluating predictors of 30-day postoperative mortality demonstrated that advanced systemic disease burden, represented by higher ASA scores, was the strongest independent predictor of mortality (OR = 11.6, *p* = 0.003). Emergency surgery was also significantly associated with increased mortality risk (OR = 7.54, *p* = 0.022), reflecting the physiologic instability and increased complexity of urgent procedures. Severe postoperative complications (Grade III–IV) and pleural breach significantly increased mortality risk, confirming that both postoperative deterioration and intraoperative technical complications substantially influence short-term survival outcomes. Smoking also remained an independent predictor of mortality, although with a more moderate effect size.

Operative and technical variables additionally influenced mortality outcomes. Nissen fundoplication demonstrated a significant protective effect compared with no fundoplication (OR = 0.26, *p* = 0.040), whereas Dor and Toupet fundoplication were not independently associated with mortality reduction. V-Loc suture use and Phasix mesh reinforcement were both associated with significantly lower mortality risk, while Ethibond suture use showed no significant independent association. Tissue glue use also demonstrated a modest protective effect. Furthermore, longer operative duration was significantly associated with increased mortality risk, suggesting that prolonged procedures may reflect greater operative complexity and physiological stress. Overall, the model highlights the combined impact of patient comorbidity, surgical urgency, intraoperative technical factors, and operative strategy on short-term postoperative survival.

**Table 7:** Multivariable Logistic Regression Analysis of Predictors of 30-Day Postoperative Mortality

Predictor	B	SE	Wald	*p*-value	OR (Exp(B))	95% CI
ASA Score (III–IV vs. I–II)	2.45	0.81	9.12	0.003	11.60	2.4–55.1
Smoking (Yes vs. No)	0.89	0.44	4.10	0.043	2.43	1.02–5.78
Nissen Fundoplication vs. No Fundoplication	-1.36	0.66	4.22	0.040	0.26	0.07–0.95
Dor Fundoplication vs. No Fundoplication	-0.91	0.59	2.38	0.123	0.40	0.13–1.28
Toupet Fundoplication vs. No Fundoplication	-0.63	0.61	1.07	0.302	0.53	0.16–1.77
Phasix Mesh vs. No Mesh	-1.11	0.56	3.93	0.047	0.33	0.11–0.98
Ovitex Mesh vs. No Mesh	-0.57	0.57	1.00	0.317	0.57	0.18–1.83
Procedure Duration (hours)	0.48	0.21	5.12	0.024	1.62	1.06–2.48

## Discussion

Based on the results of the retrospective comparative study carried out, it can be concluded that both robotic and laparoscopic hiatal hernia repairs are effective and safe methods of minimally invasive treatment. The demographic parameters were indicative of an average hiatal hernia patient group characterized by middle age and obesity, as well as by a high number of comorbidities and GERD symptoms. In terms of the surgical aspects of the procedure, it can be seen that laparoscopic repair is performed more often, and robotic repair is becoming more common. One of the important findings was a significantly increased operative time for the robotic procedure, which is explained by the longer learning curve and the need to dock the robot. At the same time, there was a trend towards a lower complication rate and lower recurrences in robotic surgery, but these differences were not statistically significant [8–10].

The postoperative outcomes further confirmed that the procedure for fixing hiatal hernias irrespective of whether performed using a robotic system or laparoscopically was associated with low mortality rates and tolerable morbidity without any mortalities recorded within 30 days after surgery in the current population. The overall recurrence rate of 19.8%, especially in the first postoperative year, is in line with earlier reports in the literature, and the significance of follow-up can be clearly emphasized here. Multivariate analysis showed that the predictors for recurrence and unfavorable outcomes were high ASA scores, emergency surgeries, smokers, pleural tear, and serious complications after surgery. However, interestingly enough, some technical factors, including Nissen fundoplication, use of V-Loc sutures, mesh augmentation, and application of tissue glue, were found to offer protection against recurrence. In general, although robotic surgery appears to be promising, the results imply that patient selection, surgeon experience, and surgical techniques may play a more pivotal role than the surgical system itself [11–15].

GERD and hiatal hernias continue to be prevalent disorders often necessitating surgery, with anti-reflux surgery achieving success rates of 85–96% among patients whose condition is medically resistant [1]. The minimally invasive approach has come to be the standard treatment method, with conventional laparoscopic procedure being extensively used because of its proven safety and low rate of complications in comparison to open surgery. Nevertheless, robotic repair of hiatal hernias has also begun to gain popularity, potentially providing benefits such as increased maneuverability, enhanced visualization, and ergonomics for the surgeon. Various comparative analyses have been conducted on both procedures, some of which found shorter hospital LOS and lower incidences of postoperative complications in robotic surgery [1, 5, 6], whereas others found no improvement or even higher complications in robotic surgery [3, 7, 16–18]. Thus, this study aimed at contributing additional data on comparing the two procedures in terms of their surgical outcomes, complications, and recurrence rates.

For the current study, there was no statistically significant difference in LOS between the two types of repair. However, the robotic group had a trend for shorter recovery period than the laparoscopic repair. This result contradicts various other researches that showed significant reductions in LOS in patients undergoing robotic surgery. For instance, Soliman et al. observed that patients undergoing robotic surgery had shorter periods of hospitalization (1.3 days compared to 1.8 days) [5]. Additionally, Tolboom et al. also found a reduction in LOS in patients undergoing redo hiatal hernia repair using robotic surgery [1]. Likewise, O’Connor et al. observed a reduction in LOS in patients undergoing robotic surgery while Ward et al. observed a trend towards increased LOS [3, 6].

With respect to postoperative complications, the results of the current analysis showed that robotic repair had a lower incidence of complications and more severe complications, but these differences did not reach statistical significance. The complications most commonly identified included pneumothorax and pleural injury, which occurred more frequently in the laparoscopic repair group [19–20]. This is somewhat consistent with the study by Tolboom et al., who found pleural defects, esophageal injuries, or gastric injuries in both groups but with different incidence [1]. Likewise, Ward et al. found a higher number of cases of esophageal perforation in the robotic group, suggesting that the types of complications may differ according to surgeon experience and case difficulty [3]. On the contrary, Soliman et al. showed a lower occurrence of adverse outcomes in robotic repair, such as infectious and respiratory complications [5]. Low rates of bleeding and infections were also found in the current analysis, corroborating the results of other published studies showing similar complication profiles in both approaches [3, 5]. Dysphagia was not observed as a postoperative complication in the current study, differing from other reports indicating low levels of postoperative dysphagia in both groups [1, 4].

In regard to the use of the ICU, our findings did not reveal any statistically significant differences between the groups, though there were some indications that the robotic repair led to a greater number of ICU admissions and the laparoscopic repair involved a longer ICU stay. This is in line with the findings of Soliman et al., who found no significant difference in ICU admissions between the laparoscopic and robotic repair [5]. Furthermore, Ward et al. observed some variations in respiratory complications; however, there was an absence of clear definition of ICU-level requirements [3].

Lastly, there were no mortalities within the 30-day period in any of the two groups of patients in our study, similar to what is seen in most published studies, with an almost negligible mortality rate for both laparoscopic and robotic hiatal hernia repairs [1, 5]. Mortalities were only sporadic in larger retrospective studies without any significant difference between the two techniques [3, 4]. It is evident from all these findings that both laparoscopic and robotic hiatal hernia repairs are relatively safe surgical procedures with very good short-term survival rates [20–24].

Among the strengths of this study, include a relatively large number of subjects in a single-center retrospective study, complete analysis of short- and mid-term results, including surgical data, postoperative complications, and recurrences. The use of multivariate logistic regression analysis adds strength to the results by determining independent factors associated with complications while controlling for confounders. Moreover, the study reflects practical surgical experience, thus improving external validity by incorporating a wide range of patients, operative procedures, and postoperative care experiences. On the contrary, the main weaknesses of the study include its retrospective design, which makes it prone to various biases, such as selection bias, information bias, and incomplete data. In addition, the non-randomization of the allocation of subjects to laparoscopic or robotic groups poses a risk of confounding, especially due to surgeon preferences and cases. The study being conducted at a single center can also be regarded as a limitation because of generalizability issues related to varying expertise and resources of other centers. Future studies should focus on prospective, multicenter randomized controlled trials with standardized surgical protocols and longer follow-up periods to better define the true comparative effectiveness of laparoscopic versus robotic hiatal hernia repair. Additionally, future research should incorporate patient-reported outcomes, cost-effectiveness analysis, and quality-of-life measures to provide a more comprehensive assessment of both surgical approaches.

## Conclusion

In this retrospective comparative study, both laparoscopic and robotic hiatal hernia repair demonstrated acceptable short-term safety and low rates of readmission, reoperation, and mortality. Robotic repair was associated with a significantly longer operative time but a shorter postoperative hospital stay, while recurrence was significantly more frequent following laparoscopic repair. Several patient-, disease-, and procedure-related factors, including higher ASA score, smoking, emergency surgery, severe postoperative complications, and operative duration, were independently associated with recurrence. Given the retrospective, non-randomized design and potential for residual confounding, these findings should be interpreted as associations rather than evidence of causal superiority, and prospective studies with longer standardized follow-up are warranted to confirm the comparative effectiveness and long-term durability of the two approaches.

## Data Availability

The datasets generated and/or analyzed during the current study are not publicly available because they contain confidential patient information and are subject to institutional and ethical restrictions. De-identified data may be made available from the corresponding author on reasonable request, subject to approval by the relevant institutional review board and the participating institution.
